# Reconstitution of T follicular helper-humoral immune axis with elimination of hepatitis C virus

**DOI:** 10.1038/s41598-020-77020-2

**Published:** 2020-11-16

**Authors:** Arshi Khanam, Shyamasundaran Kottilil, Eleanor Wilson

**Affiliations:** Division of Clinical Care and Research, Institute of Human Virology, University of Maryland, School of Medicine, 725 West Lombard Street, S218, Baltimore, MD 21201 USA

**Keywords:** Lymphocyte activation, Hepatitis C

## Abstract

Exhaustion of Hepatitis C Virus (HCV)-specific T cells and abnormal B cell function is a hallmark of chronic HCV infection. Direct-acting antiviral (DAA) therapies are effective in achieving sustained virologic response (SVR), however, whether successful DAA treatment reconstitute T follicular helper (T_FH_)-B cell axis in HCV patients is unclear. Here, we aimed to evaluate the immunological changes in global and HCV-specific CD4 + CXCR5 + T_FH_, CD4 + CXCR5-T and B cells in 20 HCV patients who achieved SVR with Sofosbuvir and Ledipasvir for 12 weeks and compared with 15 healthy controls (HC). Global and HCV-specific CD4 + CXCR5 + T_FH_, CD4 + CXCR5-T and CD19 + B cells had significant phenotypic and functional reconstitution post DAA therapy. Reconstitution of effector, central and terminally differentiated memory cell population and increased ICOS and BCL6 expression was seen in HCV patients at SVR12. HCV-specific cytokines were also improved post DAA. Exhausted and regulatory B cells were declined whereas memory B cells were expanded post DAA therapy. Importantly, frequencies of T_FH_ cells were significantly associated with HCV RNA reduction, expansion of memory B and plasmablasts, while negatively associated with exhausted/regulatory B cells. Our results demonstrate that SVR with DAA therapy is effective in the reconstitution of phenotypic and functional abnormalities of T_FH_-B cell axis.

## Introduction

Hepatitis C virus infection (HCV) is a global health burden, affecting approximately 71 million people worldwide^1^. Chronic HCV infection may lead to cirrhosis and hepatocellular carcinoma which is associated with high mortality in these patients^2^. HCV persistence may be attributed to specific defects in innate and adaptive immune responses^3^. Chronic infection leads to prompt exhaustion of CD4 T cells^4^ characterized by an increased programmed death-1 (PD-1), cytotoxic T-lymphocyte associated protein 4 (CTLA-4) expression and reduced effector cytokines including IL-21, IFN-γ and TNF-α^5–7^. Lower T follicular helper (T_FH_) cell frequency and functionality is associated with impaired humoral response and uncontrolled virus replication, suggesting crucial involvement of T_FH_ cells in governing viral infection^8^. During chronic HCV infection, decreased frequency of circulating IL-21 producing T_FH_ cells has been reported^9^. HCV-specific IL-21 secreting T_FH_ cells are critical for HCV viral control in HIV/HCV coinfection^10^. Conversely, HCV patients with cyroglobulinemic vasculitis display higher frequencies of IL-21 producing T_FH_ cells that contributed to aberrant B cell activation and generation of pathogenic IgG and IgM with rheumatoid factor activity^11^. These findings demonstrate contrasting behaviour of T_FH_ cells in HCV patients with and without cyroglobulinemic vasculitis.

Significant alterations in B cell compartment have been reported during chronic HCV infection. Although, the frequencies of circulating B cells do not alter, but the prevalence of activated B cells has been observed, especially in memory cell compartment that correlate with HCV viral load^12^. HCV patients with cyroglobulinemic vasculitis displayed higher frequencies of autoreactive memory B cells that declined after DAA therapy^11^. Interestingly, memory cell compartment also exhibited higher expression of exhaustion marker Fc receptor-like 4 (FcRL4) in HCV patients in comparison to healthy controls; however, that represent a mechanism of defense against deleterious effects of a persistent hyperactive environment in HCV patients^13^. HCV also up regulate B cell receptor signaling and associate with B cell-lymphoproliferative disorders^14^.

The introduction of highly effective interferon-free direct-acting antiviral (DAA) treatments caused a paradigm shift in HCV treatment, helping many more patients achieve clinical cure than interferon-based therapies. DAA treatments are pan-genotypic, inhibiting key HCV life cycle proteins, and when used in multiple combinations, produce sustained virological response (SVR) rates approximating 99%, with shorter treatment duration (12 weeks) and minimal side effects. Emerging data for DAA treatment support a quick and complete restoration of most innate immune cells in the blood as well as hepatic parenchyma with resolution of liver inflammation in HCV patients^15–17^. However, inadequate data is available about the reconstitution of adaptive immunologic response after DAA therapy. Besides, whether successful DAA treatment will improve T_FH_ and B cell response in HCV patients, which could contribute in viral clearance, is not yet clear. Therefore, in the present study, we aimed to evaluate if clearance of HCV infection following DAA therapy results in reconstitution of T_FH_ and B cell phenotype and function. To investigate, CD4 + CXCR5 + T_FH_ cells and CD4 + CXCR5- T cells were studied for phenotypic alterations, virus-specific and global cytokine response. Reversal of B cell abnormalities was examined. Our results indicate that SVR after DAA therapy efficiently improves the abnormalities in phenotype and function of CD4 + CXCR5 + T_FH_ cells, CD4 + CXCR5- T cells and B cells.

## Results

### Characteristics of HCV patients

HCV patient’s baseline characteristics are detailed in Table1. Out of 20, 11 patients (55%) were chronically infected with HCV genotype 1a and 9 with genotype 1b (45%). Baseline viral load was high (median-2.1 × 10^6^, range-7 × 10^4^–1.2 × 10^7^) which decreased drastically on therapy and remained undetected at SVR12. Clinical parameters of HCV patients pre and post DAA therapy are shown in Table 2. At baseline, HCV patients had elevated alanine transaminase (ALT) and aspartate transaminase (AST) levels, which normalize at SVR12. Direct bilirubin level was higher at baseline and decreased significantly at SVR12.

**Table 1:** Baseline characteristics of study subjects.

Characterstics	Healthy Controls (HC) (n = 15)	HCV (n = 20)	P value
Age (Years)	45 (27–63)	59(32–64)	NS
**Gender**			
Male	6	14	NS
Female	8	6	NS
**Race (n)**			
African Americans	10	14	NS
White	4	6	NS
**Ethnicity**			
Hispanic	2	1	NS
Non-Hispanic (n)	12	19	NS
Genotype 1a/1b (n)	NA	11/9	NA
Liver Fibrosis (F0-F2/F3-F4) (n)	NA	13/7	NA
Viral load (IU/mL) (at baseline)	NA	2.1 × 10^6^ (7 × 10^4^ – 1.2 × 10^7^)	NA

NA, Not applicable; NS, Non-significant.

**Table 2:** Clinical characterstics of the HCV patients pre and post DAA therapy.

Patient characterstics	Baseline	SVR12	P value
ALT (U/L)	82.1 (33–224)	25.7 (16–46)	< 0.0001
AST (U/L)	74.4 (30–221)	25.5 (9–41)	< 0.0001
Total bilirubin (mg/dL)	0.66 (0.3–1.3)	0.52 (0.3–1.1)	0.10
Direct Bilirubin	0.26 (0.1–0.6)	0.13 (0.1–0.3)	0.0005
Albumin (g/dL)	3.9 (3.3–4.6)	4.1(3.7–4.6)	0.12
Platelets (UL)	173 (69–305)	174 (88–249)	0.95

Values are presented as median (range) and number.

ALT, Alanine aminotransferase; AST, Aspartate aminotransferase. NA, Not applicable.

### Reconstitution of global and HCV-specific CD4 + CXCR5 + T_FH_ and CD4 + CXCR5- T cell phenotypes in HCV patients after elimination of hepatitis C with DAA treatment

**Figure 1:**
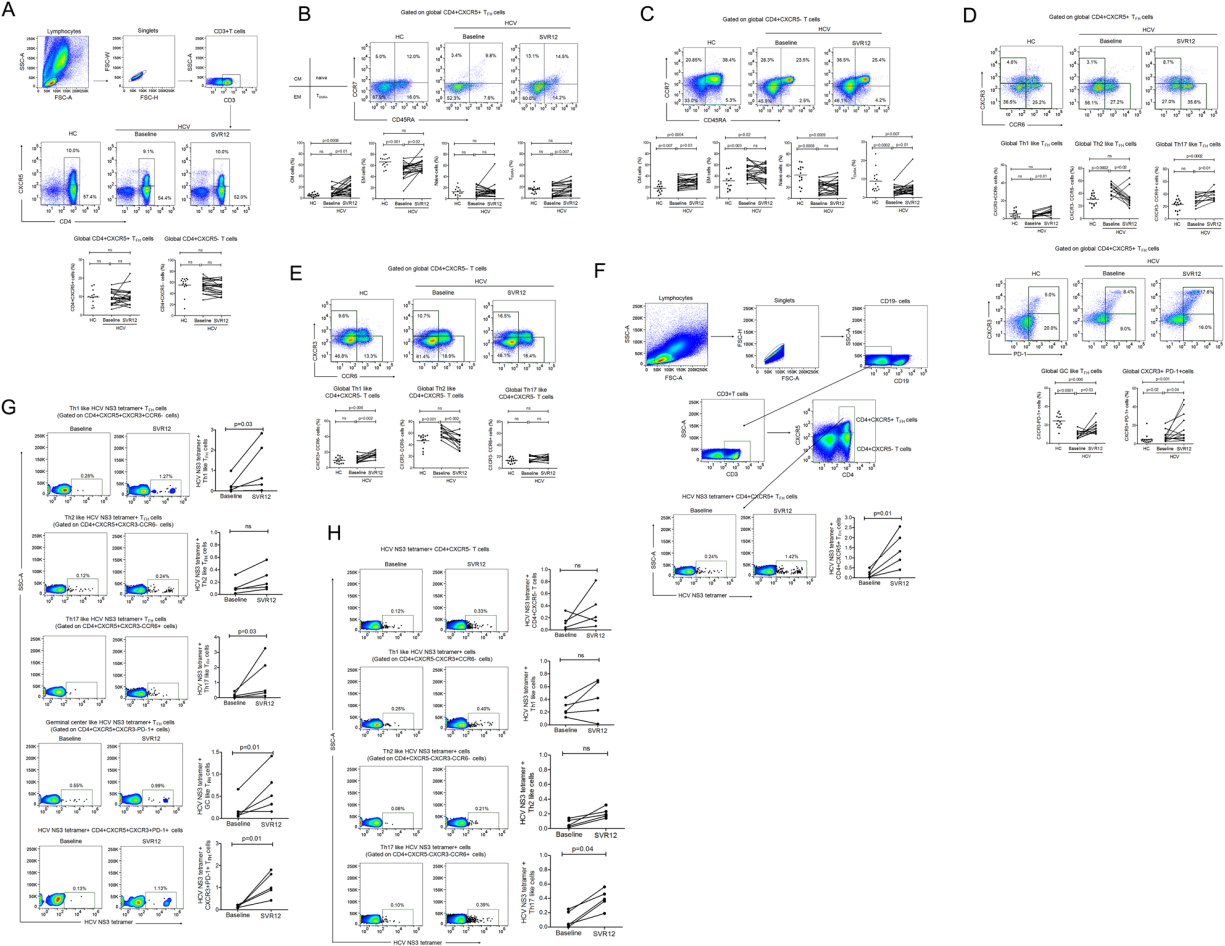
Improved global and HCV-specific CD4 + CXCR5 + T_FH_ and CD4 + CXCR5- T cell phenotypes in HCV patients after elimination of hepatitis C with DAA treatment. (**A**) Comparisons of the frequencies of CD4 + CXCR + T_FH_ cells and CD4 + CXCR5- T cells in HC (n = 15) and HCV patients (n = 20) pre and post DAA therapy. To define CD4 + CXCR + T_FH_ cells and CD4 + CXCR5- T cells, gates were first set on lymphocytes and then CD3 + T cells were gated and frequencies were analysed (**B**,**C**) Expansion of central memory (CM) effector memory (EM) and terminally differentiated effector memory cells (T_EMRA_) in HCV patients after DAA treatment. Representative flow cytometry plot and line graphs dipicts different subset of CD4 + CXCR + T_FH_ cells and CD4 + CXCR5- T cells based on the expression of CCR7 and CD45RA (CD45RA-CCR7- cells: EM, CD45RA-CCR7 + : CM, CD45RA + CCR7- : T_EMRA_ and CD45RA + CCR7 + : naïve cells). (**D**) Comparative analysis of different subset of global CD4 + CXCR5 + T_FH_ cells including Th1, Th2, Th17, GC like cells and CD4 + CXCR5 + PD-1 + CXCR3 + cells in HC and HCV patients pre and post DAA therapy. (**E**) Frequencies of global CD4 + CXCR5- T cell subsets (**F**) Representative flow cytometry plot and further line graphs indicate the frequencies of HCV-specific, NS3 tetramer-positive CD4 + CXCR + T_FH_ cells, and (**G**) Th1, Th2, Th17 GC like and CD4 + CXCR45 + CXCR3 + PD1 + T_FH_ cells (**H**) Percent frequencies of HCV-specific CD4 + CXCR5- T cells and their subsets in HCV patients (n = 5) at baseline and SVR12. Unpaired T test or Mann Whitney test was used to analyse statistical differences between HC and HCV patients. Paired T test or Wilcoxon matched-pairs signed rank test was used to calculate p values in HCV patients pre and post DAA therapy. Ns: non-significant.

CD4 + CXCR5 + T_FH_ cells are specialized in T cell mediated B cell help and are essential for germinal center (GC) formation, development of memory B cells and high affinity antibodies which remain the basis of long term protective humoral response. We observed comparable frequencies of global CD4 + CXCR5 + T_FH_ cells between HC and HCV patients that did not change after HCV elimination post DAA treatment. This indicates that neither HCV infection nor DAA treatment induces any alterations in the frequencies of CD4 + CXCR5 + T_FH_ cells. We also analysed global CD4 + CXCR5- T cells and similar data was obtained (Fig. 1A). Moreover, to investigate if HCV eradication had any influence on different subsets of CD4 + CXCR5 + T_FH_ cells and CD4 + CXCR5- T cells, the frequencies of central memory (CM), effector memory (EM), naïve and terminally differentiated effector memory (T_EMRA_) cells were analysed. Compared with HC, baseline HCV samples presented lower frequencies of EM cells, while no change in CM, naïve and T_EMRA_ cells. CM, EM and T_EMRA_ were significantly increased with HCV clearance post DAA treatment; however, naïve cells remained comparable (Fig. 1B). When CD4 + CXCR5- T cell population was analysed, baseline HCV samples displayed higher frequencies of CM and EM cells, while lower frequencies of naïve and T_EMRA_ cells. T_EMRA_ cells were increased and CM cells were further expanded post DAA therapy (Fig. 1C). Additionally, we checked for Th1, Th2 and Th17 like subset of T_FH_ cells. The frequencies of Th1 and Th17 like T_FH_ cells were lower in baseline HCV samples and increased significantly at SVR12, while the percentage of Th2 like T_FH_ cells declined significantly post DAA therapy. Another subset of circulating memory T_FH_ cells, defined as CD4 + CXCR5 + CXCR3-PD-1 + cells that have similarities with GC T_FH_ cells and are highly functional in terms of B cell help were significantly lower in HCV patients before DAA treatment and recovered at SVR12, but did not completely normalize. In addition, we analysed CD4 + CXCR5 + CXCR3 + PD-1 + cells that were higher in HCV patients and further expanded at SVR12 (Fig. 1D). Similarly, any alterations in different subsets of CD4 + CXCR5- T cells were also analysed. Percent frequencies of Th1 like CD4 + CXCR5- T cells increased, whereas Th2 like subset decreased post DAA therapy; however, no change was seen in Th17 like subset (Fig. 1E).

During chronic HCV infection, mostly global CD4 + CXCR5 + T_FH_ cells have been studied and our data did not reveal any change in the frequencies of global T_FH_ cells. Therefore, to investigate whether there was any reconstitution in HCV-specific T_FH_ cells that may associate with antiviral immunity; we assessed the differences in HCV-specific, tetramer-positive T_FH_ cells in HCV patients pre and post DAA therapy. Our results showed that the frequencies of HCV NS3 tetramer-positive + T_FH_ cells were lower in HCV patients at baseline and tended to increase at SVR12 (Fig. 1F). Moreover, frequencies of HCV-specific Th1, Th17 and GC like T_FH_ cells and CD4 + CXCR5 + CXCR3 + PD-1 + T_FH_ cells was decreased in HCV patients at baseline and reconstituted significantly at SVR12; however, Th2 like cells remain comparable pre and post DAA therapy (Fig. 1G). Frequencies of HCV-specific CD4 + CXCR5- T cells and their subset including Th1 and Th2 did not change after DAA therapy; except for Th17, which increased significantly post DAA treatment (Fig. 1H) Collectively, our data suggest that DAA therapy resuscitate global as well as HCV-specific T_FH_ cells and its subtypes. Improvement of HCV-specific phenotypes might help in viral clearance and provide immunity against reinfection.

### Upregulation of BCL6 and ICOS on global and HCV-specific CD4 + CXCR5 + T_FH_ cells post DAA therapy

**Figure 2:**
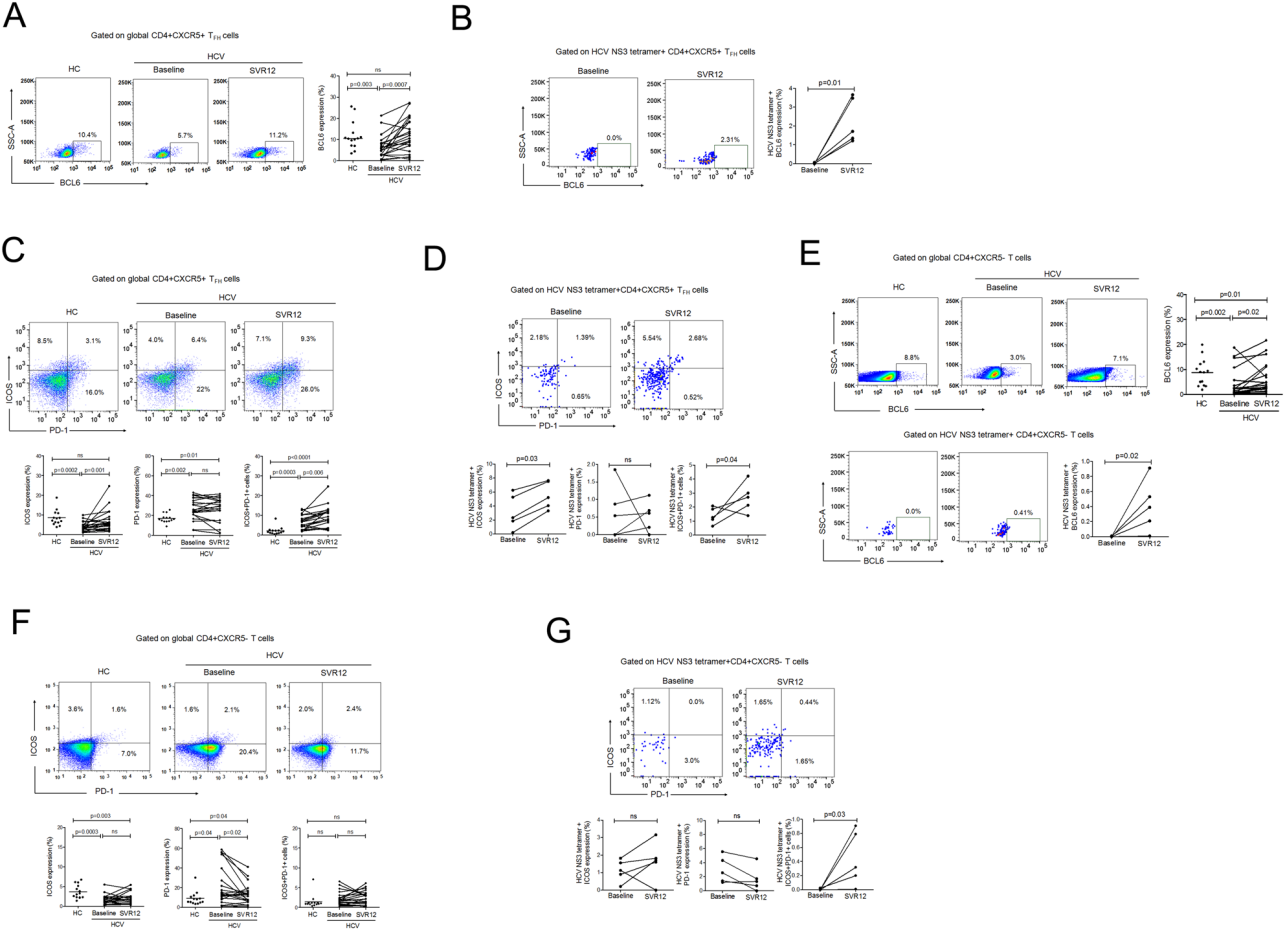
Improvement in BCL6 and ICOS expression on global and HCV-specific CD4 + CXCR5 + T_FH_ cells post DAA Therapy. (**A**–**G**) Representative flow cytometry plot and further line graphs illustrating the expression of BCL6, ICOS, PD-1 and ICOS + PD1 + cells on global CD4 + CXCR5 + T_FH_ cells and CD4 + CXCR5- T cells in HC (n = 14) and HCV patients (n = 20) and HCV-specific cells (HCV-n = 5) before and after DAA therapy. Analysis between HC and HCV patients was done using unpaired T test or Man Whitney test. Paired T test or Wilcoxon matched-pairs signed rank test was used for comparisons between HCV patients pre and post DAA treatment.

B-cell lymphoma 6 (BCL6), a transcriptional repressor is indispensable for the formation of CD4 + CXCR5 + T_FH_ cells and GC B cells^18^; mice deficient in BCL6 fail to develop GC^19^. We noticed that HCV patients had significantly lower expression of BCL6 on global and HCV-specific T_FH_ cells at baseline, which improved at SVR12 (Fig. 2A,B). The process of CD4 + CXCR5 + T_FH_ cells mediated differentiation and maturation of B cells into high-affinity memory B cells and antibody producing long-lived plasma cells is regulated by ICOS^20^. ICOS knock-out/ICOS-deficient mice had very few CD4 + CXCR5 + T_FH_ cells and very small GC^21^, that resulted in severely compromised antigen-specific immunoglobulin. Our data demonstrated that before DAA treatment HCV patients had significantly lower ICOS expression but that increased and normalized by SVR12. Generally, high PD-1 expression on T cells has been shown to be associated with functional exhaustion^22,23^. Contrarily, CD4 + CXCR5 + T_FH_ cells expressing high PD-1 considered being highly functional^24^. HCV patients showed higher PD-1 expression on CD4 + CXCR5 + T_FH_ cells than HC, which maintained high even at SVR12. Co-expression of ICOS and PD-1 was also higher in HCV patients, which further boosted at SVR12. ICOS and ICOS + PD-1 co-expression augmented on HCV HCV-specific T_FH_ cells; however, PD-1 alone did not change post DAA therapy (Fig. 2C,D).

Similarly, expression of BCL6, ICOS and PD-1 was also analysed on global and HCV-specific CD4 + CXCR5- T cells. In baseline HCV samples, global CD4 + CXCR5- T cells showed poor BCL6 and ICOS while higher PD-1 expression than HC. BCL6 expression amplified whereas PD-1 declined significantly at SVR12; however, no change in ICOS expression was seen. Decline in PD-1 on CD4 + CXCR5- cells may lead to the functional restoration of cells that could help in viral clearance. The analysis of HCV-specific CD4 + CXCR5- T cells revealed higher BCL6 and ICOS + PD-1 co-expression after DAA treatment; though, expression of ICOS and PD-1 did not change significantly (Fig. 2E-G). Together, these data unveil substantial changes in immunophenotypic profile of global as well as HCV-specific cells after DAA therapy.

### Reconstitution of HCV-specific cytokines post DAA treatment

**Figure 3:**
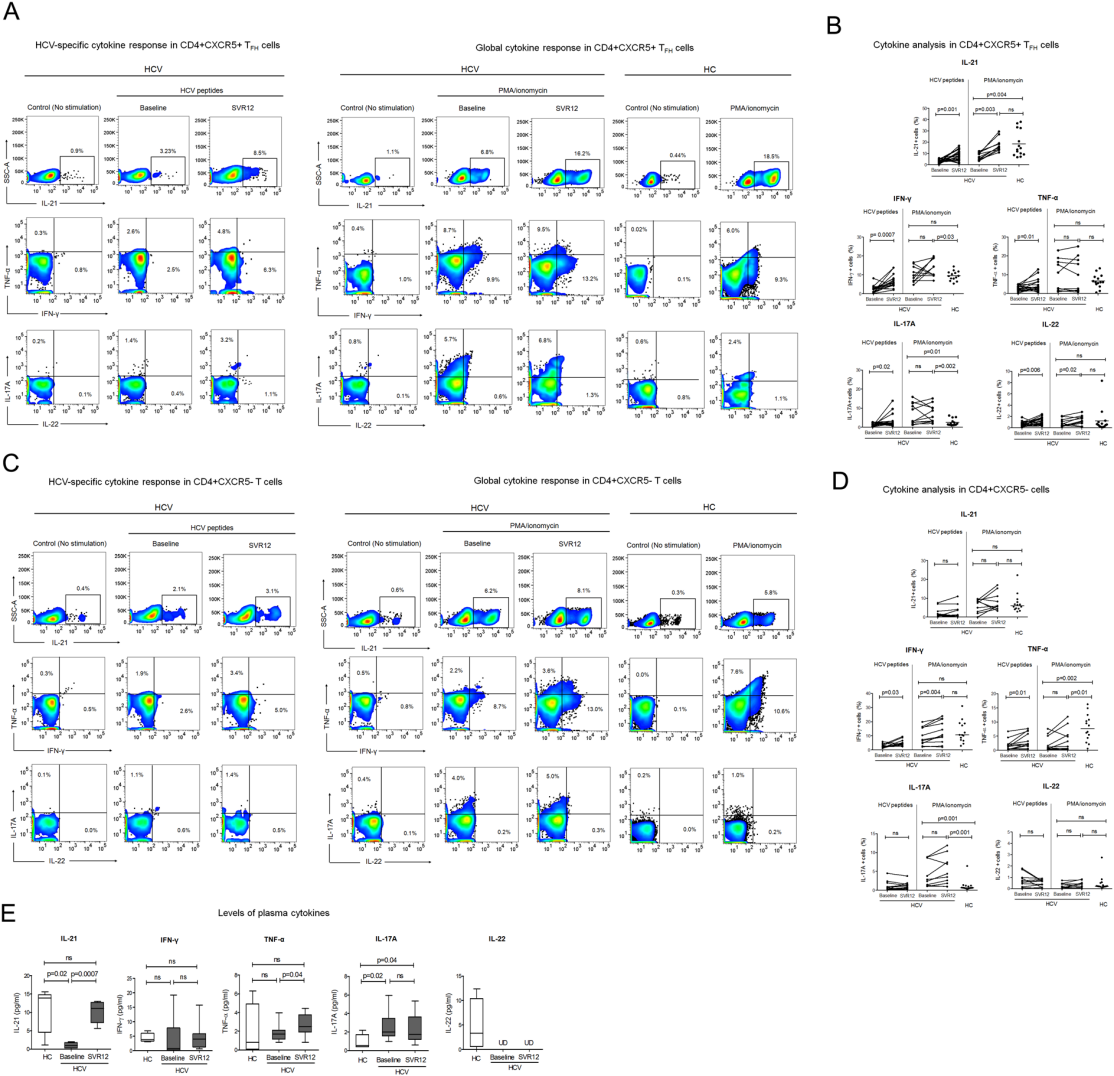
Augmentation of HCV-specific cytokine production following treatment with DAA**.** To analyse the HCV-specific cytokine production, PBMCs were stimulated with HCV peptides spanning the entire HCV genome in the presence of CD49d and CD28 for 5 days and followed by surface staining for CD4 + CXCR5 + T_FH_ cells and CD4 + CXCR5- T cells and intracellular staining for cytokine detection. To measure global cytokine production, PBMCs were stimulated with PMA/ionomycin for 18 h. (**A**,**C**) Representative flow cytometry images illustrating HCV-specific and global cytokine response by CD4 + CXCR5 + T_FH_ cells and CD4 + CXCR5- T cells and (**B**,**D**). Cumulative data has been presented in line graphs for HCV patients and scatter dot plot for HC. (**E**) Levels of plasma cytokines were observed by multiplex cytokine bead array assay. P values were evaluated by unpaired T test or Mann Whitney test for comparisons between HC and HCV. For analysing data in HCV patients pre and post DAA therapy, Paired T test or Wilcoxon matched-pairs signed rank test was used. HCV-specific cytokine production was analysed in 20 HCV patients at both time point, while global cytokine response was examined in 11 HCV patients and 14 HC individuals.

To find out whether improved CD4 + CXCR5 + T_FH_ cell phenotype ameliorate the functional ability of these cells, we analysed HCV-specific as well as global cytokine secretion. Our data established significant augmentation of HCV-specific cytokines including IL-21, IL-17A, IL-22, IFN-γ and TNF-α by CD4 + CXCR5 + T_FH_ cells in HCV patients at SVR12. Global IL-21 production was also compromised in HCV patients, which improved after DAA therapy. Production of IL-22 and IFN-γ was also enhanced; however, others did not change (Fig. 3A,B). Since PMA/ionomycin may decrease CXCR5 expression on T_FH_ cells, therefore we additionally checked global cytokine production in sorted CD4 + CXCR5 + T_FH_ cells; however, results were similar to total PBMCs (Supplementary Fig. 1A,B).

Cytokine production was also analyzed in CD4 + CXCR5- T cells. HCV-specific IFN-γ and TNF-α production was enhanced in HCV patients at SVR12. When we looked at the global cytokine response, only IFN-γ production was amplified at SVR12. When comparisons were made between HC and HCV patients, HCV patients showed significant increase in baseline IL-17A but decrease in TNF-α and this cytokine pattern did not change even at SVR12. Other cytokines were comparable in HC and HCV patients. (Fig. 3C,D).

Plasma cytokines were also detected. In comparison to HC, HCV patients had significant lower level of IL-21, which recovered at SVR12. IL-17A was consistently higher in HCV patients at baseline as well as SVR12. IFN-γ and TNF-α levels were comparable between HC and HCV patients, TNF-α increased at SVR12; however, IFN-γ did not change (Fig. 3E).

### Significant reduction in exhausted and immunosuppressive B cells, while expansion of memory B cells post DAA treatment

**Figure 4:**
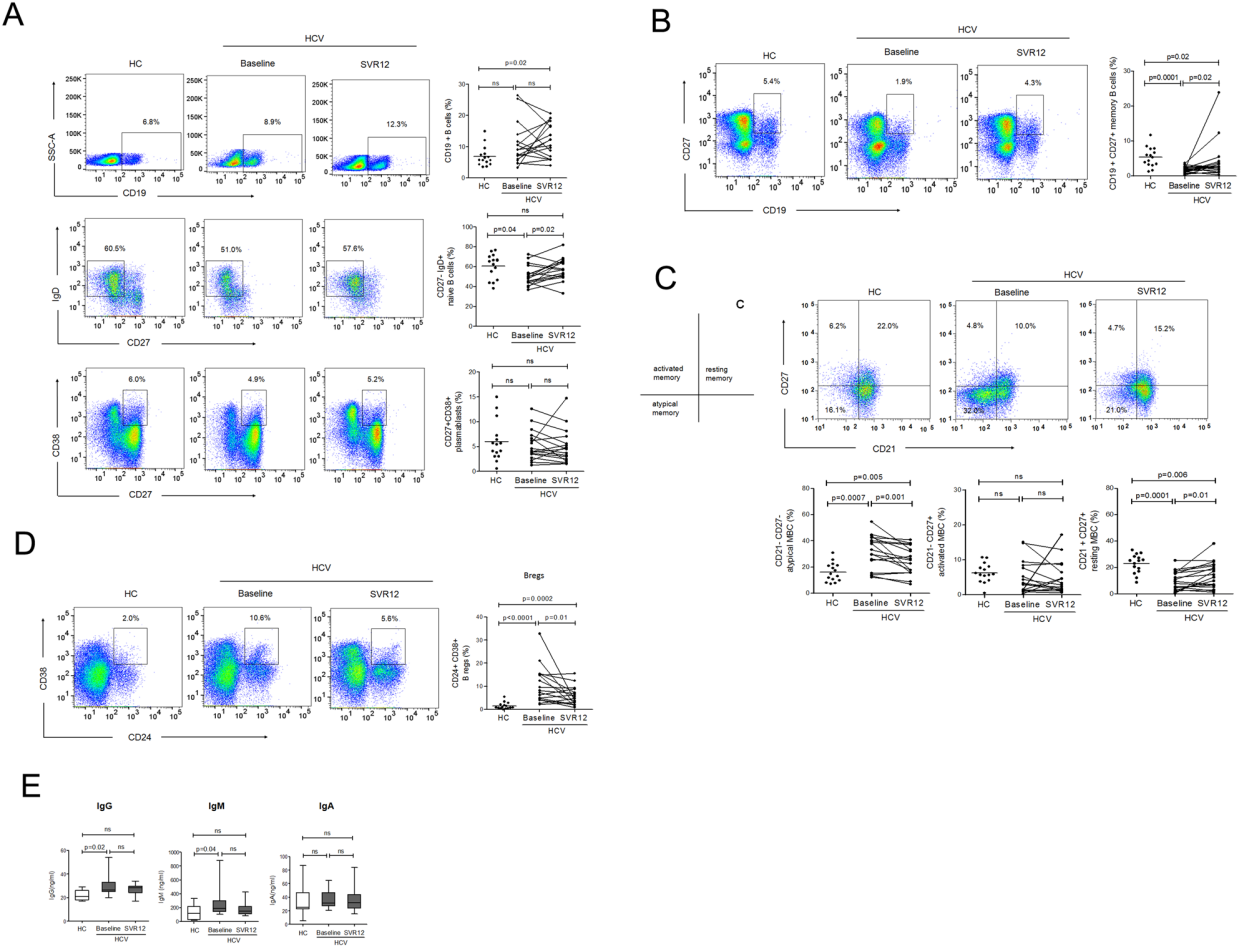
Expansion of memory B cells whereas reduction in exhausted and immunosuppressive B cells with DAA mediated viral clearance. (**A**) Representative flow cytometry plot and graphs indicate the frequencies of total B cells, naïve B cells and plasmablasts, (**B**,**C**) Memory B and its subsets and (**D**) Bregs in HC (n = 14) and HCV patients pre and post DAA therapy (n = 17). (**E**) Levels of B cells antibodies including IgG, IgM and IgA in the plasma of HC (n = 7) and HCV patients (n = 12). Mann Whitney test was used to compare HC with HCV patients. Wilcoxon matched-pairs signed rank test was used to analyse the statistical difference in HCV patients at baseline and SVR12.

We tested whether frequencies of total B cells and its subsets changes after DAA therapy. We found comparable frequencies of total B cells in HC and HCV patients; however, HCV patients displayed lower frequencies of naïve B cells at baseline that recovered at SVR12. Plasmablasts remained comparable between HC and HCV patients (Fig. 4A), while memory B cells were significantly altered in HCV: at baseline, HCV patients had lower frequencies of memory B cells which expanded at SVR12 (Fig. 4B). Memory B cell populations contained higher proportion of atypical memory B cells, an exhausted and aged phenotype, and lesser resting memory cells which reversed at SVR12 displaying clear decrease in atypical memory while increase in resting memory cells. Activated memory cells remained comparable between HC and HCV samples, at baseline and SVR12 (Fig. 4C).

Regulatory B cells (Bregs) are critical regulators of immune responses in chronic viral infections^25^, autoimmune diseases^26^ and cancers^27^. They suppress the function of effector T cells by reducing IFN-γ and TNF-α production. Previous data reported that CD19 + CD24hiCD38hi B cells promote Tregs, suppress Th1 and effector function of other T cells^28^ and are associated with disease flares in chronic hepatitis B infection^29^, depletion of which resulted restoration of effector T cell functions^30^. These findings confirm potential immunosuppressive role of Bregs during viral infection. However; in HCV infection, the status of Bregs and whether DAA treatment has any impact on Bregs remains poorly described. Our result showed that HCV patients had noticeably higher frequencies of Bregs, which declined significantly by SVR12 (Fig. 4D). This data suggests that reduction in Bregs could associate with improved CD4 + CXCR5 + T_FH_ and CD4 + CXCR5- T cell response post DAA therapy.

Humoral immunity, specially secreted neutralizing antibodies have great importance to eliminate viral infections and protect the body against these infections. Therefore, we analysed B cell antibodies including IgG, IgM and IgA in the plasma of HCV patients pre and post DAA therapy and compared with HC. At baseline, HCV patients had higher IgG and IgM antibodies that remained higher at SVR12. No change in IgA level was between HC and HCV patients (Fig. 4E). Elevated IgG and IgM level in HCV patients could be associated with DAA therapy mediated viral clearance.

### Improvement in homing receptor CXCR4 and BLIMP-1 following DAA Therapy

**Figure 5:**
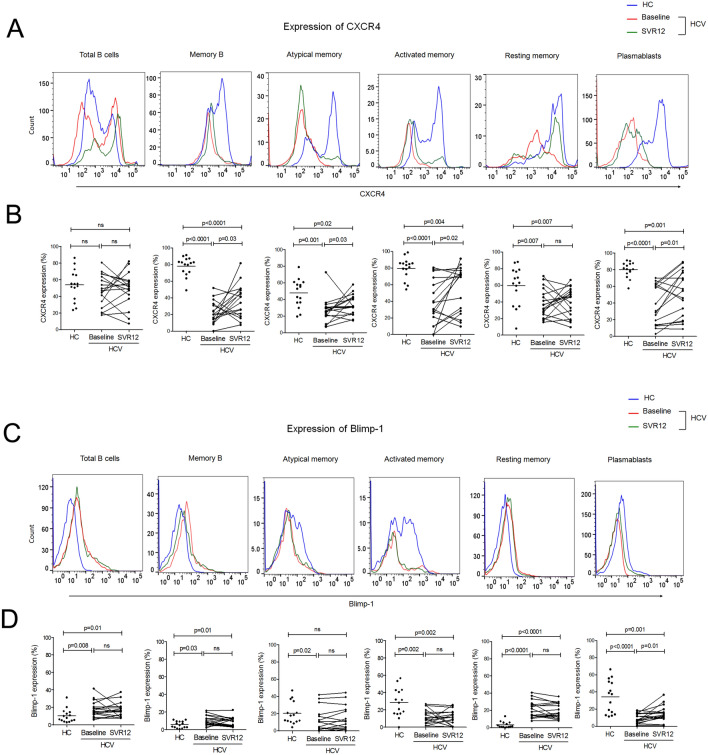
Amelioration of CXCR4 and BLIMP-1 with DAA mediated viral clearance. (**A**,**C**) Representative histograms showing the expression of CXCR4 and BLIMP-1 on different B cell subsets in HCV patients at baseline and SVR12 and HC. (**B**,**D**) Cumulative data has been presented in scatter dot plot for HC (n = 15) and by line graphs for HCV patients (n = 17). Comparisons between HC and HCV patients were performed using unpaired T test or Mann Whitney test. Statistical differences in HCV patients pre and post DAA therapy were determined by Paired T test or Wilcoxon matched-pairs signed rank test was used.

Chemokine receptor CXCR4 is expressed throughout B cell development but possesses different functions dependent on the developmental stage. It plays an essential role in B cell maturation and trafficking, regulates homeostasis of B cell compartment and humoral immunity^31^. Our data revealed that all B cell subsets including total B, memory and its subtypes comprising atypical, activated and resting memory along with plasmablasts expressed CXCR4 in HCV patients; however, expression was significantly lower than HC. The expression of CXCR4 was markedly enhanced on memory B and its subsets including activated and atypical memory as well as plasmablasts; whereas total B and resting memory cells did not show any change in CXCR4 expression at SVR12 (Fig. 5A,B).

Blimp-1 is critical for the development of immunoglobulin secreting cells as well as maintenance of long-lived plasma cells. In compression to HC, HCV patients showed higher Blimp-1 expression on total B, memory B including resting memory which remained constantly higher at SVR12. Whereas other memory subset consisting atypical and activated memory express lower Blimp-1 at baseline and maintained similar profile at SVR12. In comparison to HC, HCV patients displayed lower Blimp-1 expression on plasmablasts, which recovered after DAA therapy (Fig. 5C,D).

### CD4 + CXCR5 + T_FH_ cells are associated with B cells and HCV viral load reduction

**Figure 6:**
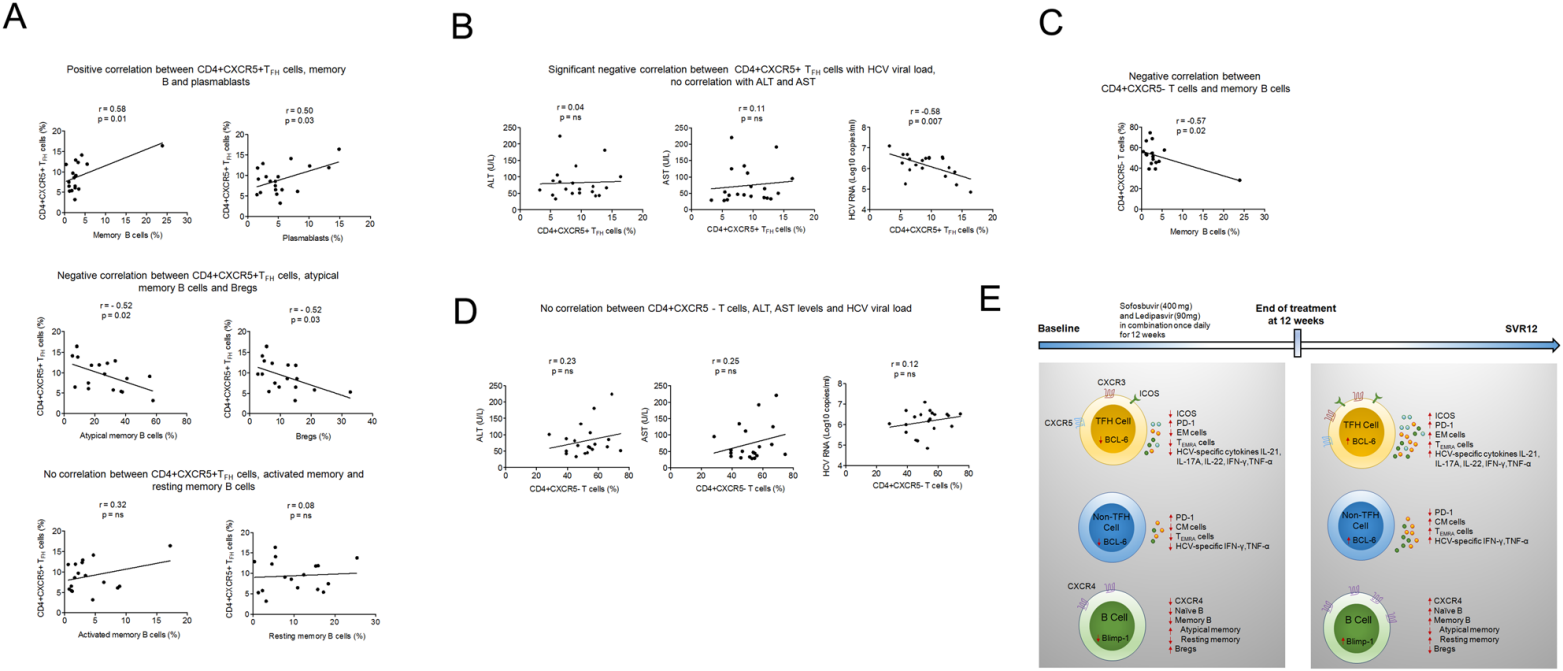
Association of CD4 + CXCR5 + T_FH_ cells with B cells and HCV viral load reduction. (**A**) Statistical correlation of the baseline frequencies of CD4 + CXCR5 + T_FH_ cells with B cell subsets including memory B and its subset, plasmablasts and Bregs. (**B**) Correlation between CD4 + CXCR5 + T_FH_ cells with HCV RNA, ALT and AST levels. (**C**) Correlation between CD4 + CXCR5- T cells with memory B cells. (**D**) between CD4 + CXCR5- T cells and HCV RNA, ALT and AST levels. Correlation was analysed using Pearson correlation coefficient. (**E**) Mechanism of reconstitution of T follicular helper-humoral immune axis with elimination of hepatitis C virus after DAA therapy. Before treatment, HCV patients had abnormal phenotype and function of CD4 + CXCR5 + T_FH_, CD4 + CXCR5- T cells and CD19 + B cells. Immune dysfunctions were reversed with eradication of hepatitis C after treatment with DAA therapy. FDC: Fixed dose combination, ICOS: Inducible T-cell co stimulator, CM; Central memory, EM, Effector memory, T_EMRA_: Terminally differentiated effector memory, PD-1: Programmed death-1, BCL6 B-cell lymphoma 6, BLIMP-1: B lymphocyte-induced maturation protein-1, CXCR: Chemokine receptor.

Next, we sought to determine whether baseline frequency of CD4 + CXCR5 + T_FH_ cells correlate with different subsets of B cells. Our results demonstrated that CD4 + CXCR5 + T_FH_ cell frequency positively correlate with memory B cells (r = 0.58 p = 0.01) and plasmablasts (r = 0.50 p = 0.03). A negative correlation was seen between CD4 + CXCR5 + T_FH_ cells, atypical memory B cells (r = -0.52 p = 0.02) and Bregs (r = 0.52 p = 0.03), whereas no correlation was seen with resting as well as activated memory B cells. Subsequently, we examined whether the frequency of CD4 + CXCR5 + T_FH_ cells associate with HCV RNA, ALT and AST levels, markers of liver inflammation in HCV patients (Fig. 6A). The results showed significant correlation between CD4 + CXCR5 + T_FH_ cells with HCV viral load reduction; however, no correlation was seen with ALT, AST levels (Fig. 6B). Moreover, CD4 + CXCR5- T cells showed correlation only with memory B cells (r = -0.57 p = 0.007) (Fig. 6C) but not with other B cell subsets. In addition, no correlation was seen between CD4 + CXCR5- T cells and clinical parameters (Fig. 6D). Results are summarized in figure (Fig. 6E).

## Discussion

Although DAA therapies are quite effective in clearing HCV infection, they do not defend from reinfection and rates of reinfection might be higher in population at risk. Protective immune response is crucial to escape from infection and provide long lasting immunity to reinfection. In this context, CD4 + T cells are of great importance. They not only empower CD8 T cell mediated response and cytotoxicity, but also facilitate B-cell mediated humoral immunity. In particular, T_FH_ cells provide help to B cells for generating humoral immune response. Although, some studies have focused on HCV-specific CD4 + T cells during acute and chronic HCV infection^32^, role of T_FH_ cells is insufficient during chronic HCV infection. Therefore, in the present study we studied both global as well as HCV-specific T_FH_ cells responses in HCV patients. We identified significant abnormalities in global and HCV-specific CD4 + CXCR5 + T_FH_ during chronic HCV infection, which may contribute to disease pathogenesis. Hence, we further investigated whether DAA therapy had any influence on these cells. Our data revealed that HCV-specific T_FH_ cells were lacking in HCV patients at baseline and significantly reinstated at SVR12. Moreover, different subtypes of HCV-specific T_FH_ cells including Th1, Th17, GC like T_FH_ cells and CD4 + CXCR5 + CXCR3 + PD-1 + T_FH_ cells were reconstituted after DAA treatment. T_FH_ molecules including BCL6, required for the establishment of T_FH_ cells and GC B cells, and ICOS, critical for T_FH_ cells mediated differentiation and maturation of B cells into high-affinity memory B cells and antibody producing long-lived plasma cells were increased after DAA therapy. The improvement in T_FH_ frequencies might be associated with increased expression of BCL6 and ICOS. The presence of more HCV-specific T_FH_ cells after the successful viral elimination might be beneficial for HCV patients as it could protect from getting chronic reinfection.

Few studies have investigated global T_FH_ cells during chronic HCV infection^9,33^. Our data did not find any change in the frequencies of global T_FH_ cells. However, irrespective of normal frequency of global T_FH_ cells at baseline, this population had a quantitative deficiency of effector cells, which reconstituted after viral clearance with DAA therapy. Although, T_FH_ cell population contained normal proportions of CM, naïve and T_EMRA_ cells; CM, and T_EMRA_ cells were increased following DAA therapy. Moreover, improvement in global T_FH_ cell activation and function was observed following DAA therapy, evidenced by augmentation of BCL6 and ICOS molecule, critical for T_FH_ cell activation, proliferation, differentiation and maintenance, GC reaction and further B cell activation. Collectively, our data suggest that global as well as HCV-specific CD4 + CXCR5 + T_FH_ cell phenotypes improved post DAA therapy that may help in DAA mediated viral clearance. A recent study has also reported the expansion of HCV-specific CD4 + T cells within two weeks after the initiation of antiviral therapy. Interestingly, these HCV-specific CD4 + T cells were polarized towards T_FH_ cells. Although the frequencies of CD4 + T cell declined toward follow-up, T_FH_ cells were maintained months after therapy-induced elimination of persistent infection, suggesting antigen-independent survival of this subset. The study further suggested T_FH_ cells as an important target population for vaccination efforts to prevent reinfection and immunotherapeutic approaches for persistent viral infection^34^. Downregulation of exhaustion and activation markers and an upregulation of memory-associated molecules were also observed after initiation of DAA therapy.

HCV patients have weak or even absent T cell response due to their exhausted phenotype^35,36^, reduced ability to proliferate^37^ and produce cytokines^38^. CD4 + CXCR5 + T_FH_ cell mediated IL-21 secretion is severely impaired in HCV patients^9^ and is associated with defective B cell proliferation and maturation^39^. Therefore, we evaluated whether improvement in CD4 + CXCR5 + T_FH_ cell phenotypes after DAA therapy had any contribution in attaining its function and we found functional restoration of CD4 + CXCR5 + T_FH_ cells after DAA therapy shown by enhanced HCV-specific and global IL-21 production. Interestingly, DAA therapy not only improved IL-21 secretion but enhanced HCV-specific IL-17A, IL-22, IFN-γ and TNF-α production, signifying that DAA therapy improves polyfunctional HCV-specific CD4 + CXCR5 + T_FH_ cell response in HCV patients. In addition, the enhancement of IFN-γ, IL-17A and IL-22 production by CD4 + CXCR5 + T_FH_ cells may indicate the expansion of HCV-specific Th1, Th17 and Th22 population within CD4 + CXCR5 + T_FH_ cells.

Generally, it is accepted that the anti-viral T cell response determines whether HCV infection will resolve or persist. In this regard, much progress has been made to classify HCV-specific T cell response. However, emerging data indicate critical involvement of humoral B cell response in providing protection against various pathogens^40–42^. B cells are crucial in inducing anti-viral T cell response by capturing and presenting specific antigens to T cells^43^. Frequencies of circulating B cells and its subpopulation are altered during chronic inflammatory disorders and are associated with disease outcome^44,45^. Particularly, during HCV infection, polyclonal B cell activation can cause cyroglobulinemia and induce arthritis^46^ and develop malignancies, such as non-Hodgkin’s lymphoma^47^. Despite the crucial role of B cell in HCV infection, they have not been extensively studied. Moreover, there is paucity of data regarding the impact of available therapeutics on different B cell compartment and functions. We found that, even though the frequency of total B cell was normal in HCV patients, there was substantial skewing of different B cell subsets. These patients had lower naïve and memory B cell population where memory cell compartment exhibited higher percentage of atypical, while lower resting memory cells. Furthermore, Bregs were drastically expanded in HCV patients. DAA therapy modulated B cell compartment by enhancing frequencies of total memory, resting memory and naïve B cells and reducing atypical memory and Bregs. Interestingly, activated memory B cells did not expand after DAA therapy, which may reduce the risk of developing cyroglobulinemia and associated co-morbidities in these patients.

CXCR4 is expressed on B cells at multiple stages of their development and fulfills different functions^31^. Migration of plasmablasts to the bone marrow niche is prerequisite of the appropriate development of long-lived plasma cells and maintenance of effective humoral immunity; this process requires CXCR4 and it has been shown that deletion of CXCR4 decreases the frequency of antibody secreting cells and the level of circulating serum immunoglobulins, hence compromising humoral immunity^31^. Expression of CXCR4 on B cell subsets have not been studied in HCV patients. We illustrated that B cell subsets express low CXCR4 in HCV patients that recovered post DAA therapy, which may contribute to appropriate functional B cell recovery in HCV patients.

Furthermore, we found a correlation between CD4 + CXCR5 + T_FH_ and B cell subsets. CD4 + CXCR5 + T_FH_ cells were positively correlated with total memory B cells and plasmablasts, while negatively correlated with atypical memory and Bregs. These observations strongly support the hypothesis that CD4 + CXCR5 + T_FH_ cells regulate the distribution of different B cell subtypes during chronic HCV infection. The findings that CD4 + CXCR5 + T_FH_ cell frequency is associated with decreased atypical memory and Bregs could be useful as atypical memory B cells are exhausted while Bregs are immunosuppressive, so the reduction in these subtypes may boost HCV-specific T cell response and contribute to viral elimination. In fact, amplification of atypical memory B cells in HIV infected individual is associated with poor antibody response against HIV infection as they do not proliferate in response to B cell receptor signalling, CD40L, CpG and TLR agonist and have decreased ability to differentiate into Ab-secreting cells^48^. Our data further revealed that frequency of CD4 + CXCR5 + T_FH_ cells correlate with reduction in HCV viral load but not with markers of liver inflammation, which may suggest that CD4 + CXCR5 + T_FH_ cells could contribute in reduction of HCV viremia without inducing liver inflammation.

Further, we studied whether DAA therapy had any effect on CD4 + CXCR5- T cells. We did not see any change is the frequencies of CD4 + CXCR5- T cells, however, CM and T_EMRA_ cells were enhanced post DAA therapy. We also observed the expansion of global Th1 like while reduction in Th2 like CD4 + CXCR5- T cells post DAA therapy; however, frequencies of HCV-specific CD4 + CXCR5- T cells and their subsets did not change post DAA treatment excluding Th17 that were enhanced. Expression of BCL6 improved while PD-1 dropped significantly post DAA therapy. Decline in PD-1 post DAA therapy may lead to functional restoration of HCV-specific as well as global cytokine responses in CD4 + CXCR5- T population, therefore we analysed cytokine secretion by these cells and found significant enhancement in HCV-specific IFN-γ and TNF-α production post DAA therapy. This indicates that DAA therapy not only improves CD4 + CXCR5 + T_FH_ cell responses but also potentially expands CD4 + CXCR5- T cell responses in HCV patients after treatment with DAA, which may further contribute to attain SVR.

Although the present study extensively demonstrated global as well as HCV-specific T_FH_ cells phenotype and function in HCV patients pre and post DAA therapy and the observations are promising, there are a few limitations. As a retrospective investigation, there was limited availability of the stored samples; therefore, only global B cell population and antibodies have been studied. In the future, it will be important to investigate the antigen-specific B cell responses in HCV patients pre and post DAA therapy. Further, to reinforce the data, characterization of class switching in HCV-specific B cells is another key area to investigate in HCV patients pre and post DAA therapy in the future. Because of limited samples available, whether DAA treatment had any influence on T_FH_ and B cell interactions in HCV patients was not studied. Additionally, the present study focused on circulating T_FH_ and B population, since it was not feasible to get lymphoid organ from HCV patients and analyse these populations in germinal centers. A better understanding of T_FH_-B cell interactions could be achieved by exploring germinal centers T_FH_ and B population, which could be included in future studies.

In conclusion, our study suggests that DAA therapy substantially improves the phenotype and functionality of these cells by enhancing HCV-specific and global cytokine response, which may considerably enhance viral clearance. Presence of the HCV-specific T_FH_ even after successful virus elimination might provide immunity to reinfection. We also showed that DAA therapy is strongly associated with improvement in the baseline abnormalities of B cell homeostasis, including lower frequencies of atypical memory B cells and Bregs, associated with defective T cell response, in HCV patients at the time of SVR. Finally, our results suggest that DAA therapy may not only effectively reduce the HCV viral load with direct antiviral effects but may also improve T and B cell functional responses.

## Patients and methods

### Subjects and treatment

In this retrospective study, we evaluated 20 HCV patients who were enrolled in the control arm of our phase 2A clinical trial number NCT01805882 between January 11, 2013 and December 17 at the clinical research centre of the National Institute of Health (NIH) Bethesda, MD USA^49^. 15 healthy controls (HC) without any history of liver disease were also taken. In HCV patients, the absence of cirrhosis was confirmed by liver biopsy or with a combination of fibroSURE test and aspartate transaminase to platelet ratio (APRI)^49^. Written and oral informed consent was received from all the participants. The key goal of HCV therapy is to achieve SVR, which is defined as the absence of detectable HCV RNA in serum 12 weeks following the completion of therapy. Therefore, Sofosbuvir (400 mg) and Ledipasvir (90 mg) tablets were given in combination once daily for 12 weeks.

### Study approval

The study protocol was approved by the institutional review board of the University of Maryland School of medicine, Baltimore, MD, USA. All the investigations were conducted as per the Declaration of Helsinki principles. Written informed consent was received from all participants.

### Samples

For phenotypic and functional immune profiling, stored peripheral blood mononuclear cells (PBMCs) and plasma samples were used from HCV patients at baseline and SVR12 and HC. Plasma was separated after centrifugation of blood at 4000 rpm for 5 min and stored at – 80 °C until use. PBMCs were isolated by density gradient centrifugation, frozen in fetal bovine serum containing 10% dimethyl sulfoxide and stored at −140 °C in liquid nitrogen.

### HCV RNA quantification

We quantified serum HCV RNA concentrations using COBAS TaqMan HCV RNA assay, version 2.0 (Roche Diagnostics, Indianapolis, IN, USA), with a lower limit of quantification of 43 IU/mL and a lower limit of detection of 15 IU/mL.

### Flow cytometry

Frozen PBMCs from HCV patients (Baseline and SVR12) and HC were thawed using complete Rosewell Park Memorial Institute 1640 medium (RPMI-1640, Sigma-Aldrich) containing 10% FBS, (Gibco), 2 mM l-glutamine (Celgro) and 1% penicillin/streptomycin (Himedia). Cells were washed twice in complete RPMI-1640 medium at 1300 rpm for 7 min and then rested overnight. Cell count and viability was determined using trypan blue exclusion method. Cells were then used as per the experimental requirement. To determine the frequency of CD4 + CXCR5 + T_FH_ and CD4 + CXCR5- T cells, PBMCs were first surface stained with anti-human CD3AF700, CD4PerCP-Cy5.5, CXCR5BV421, CXCR3FITC, CCR6APC-Cy7 CD45RABV605, CCR7BV510, PD-1PeCy7 and ICOSPE antibodies in 96 well round bottom plate for 30 min in dark at room temperature. Cells were then washed with 1XPBS. Intranuclear staining was performed for transcription factor BCL6-APC after fixation and permeabilization of cells with eBioscience FOXp3/transcription factor staining buffer set according to the manufacturer’s instructions. For B cell analyses following antibodies were used. CD19FITC, CD27BV510, CD38PerCP-Cy5.5, IgDAF700, CD24BV605 and CXCR4APC/Cy7 and then intranuclear staining with BLIMP-1APC was done. After two PBS wash, paraformaldehyde was added (0.5%) and cells were acquired on flow cytometer (BD FACSARIA II). Data analysis was done with flowJo v10 software. Details of the antibodies have been listed in supplementary Table 1.

### Major Histocompatibility Complex Class II (MHC II) tetramer staining

HLA-DRA*01:01/DRB1*15:01 restricted HCV NS3 (GINAVAYYRGLDVSV)-PE conjugated tetramer from ProImmune were used as per manufacturer’s instructions. Briefly, 2 × 10^6^ PBMCs from HCV patients were incubated with 5 µl of HLA-DRA*01:01/DRB1*15:01 matched tetramer for 2 h at 37 °C in the dark. Cells were then washed twice with wash buffer and stained with T_FH_ cell markers including CD3AF700, CD4PerCP-Cy5.5, CXCR5BV605, CXCR3FITC, PD-1BV510 and ICOSBV421 antibodies in 96 well round bottom plate for 30 min in dark at room temperature. Tetramer can bind non-specifically to B cells, therefore CD19APC antibody was also used for staining to exclude B cells while performing analysis. Intranuclear BCL6-PE-Cy7 staining was performed as per above mentioned protocol. Cells were acquired on BD FACS ARIA and analysed by flowJo.

### Sorting of CD4 + CXCR5 + T_FH_ cells

For CD4 + CXCR5 + T_FH_ cell sorting, PBMCs from HC and HCV patients were thawed, washed with complete RPMI1640 medium and rested overnight at 37°. The following day, cells were washed twice with 1X PBS and stained with anti-human CD3 AF700, CD4 PerCP-Cy5.5 and CXCR5 BV421 to sort T_FH_ cells. Cells were sorted on BD FACSARIA II flow cytometer, collected in 2 ml FBS and further stimulation assay was performed with PMA/ionomycin and cytokines were analysed.

### Intracellular cytokine detection

To investigate the HCV-specific response in chronic HCV patients, HCV overlapping peptides were used for stimulation assays. Depending on the viral genotype (Genotype 1a or 1b) present in HCV patients, PBMCs were stimulated with either genotype 1a or 1b specific overlapping peptides (5 µg/ml) along with CD49d/CD28 (2 μg/ml) (Biolegend, San Diego, CA) in 96 well flat bottom plate for 5 days at 37 °C in 5% CO2 incubator. 1 μg/ml of Brefeldin A, (BD Biosciences, San Jose, CA) was added 12 h before the completion of incubation time. HCV genotype 1a or 1b peptides were 15–18 mer with 11 or 12 amino acid overlaps spanning the entire HCV polyprotein (BEI Resources, NIAID, NIH: peptide Array, hepatitis C virus). All the peptides were dissolved in dimethyl sulfoxide and diluted in 1X PBS. We pooled the genotype 1a or genotype 1b overlapping peptides separately, made aliquots and stored at -80 °C until use. Different conc. of HCV peptides including 1, 2 and 5 µg were used to check the immunological response and cytoxicity. Better responses were seen with 5 µg conc. without any cytoxicity, therefore 5 µg conc. was used for final experiments. For global cytokine production, PBMCs were stimulated with 500 ng/ml PMA and 1 μg/ml ionomycin for 18 h at 37° and 5% CO2. Brefeldin A was added after 2 h of incubation. Cells were then washed twice with PBS and stained with Live/dead fixable far red dead cell stain (Invitrogen, Waltham, MA) for 30 min and further staining was performed using CD3AF700, CD4PerCP-Cy5.5, CXCR5BV421, IL-17APE, IL-21AF647, IL-22PeCy7, IFN-γBV605 and TNF-αBV510.

### Multiplex cytokine bead array

Concentration of plasma cytokines including IL-21, IL-17A, IL-22, IFN-γ and TNF-α were detected and quantified by multiplex cytokine bead array assay (Invitrogen, Waltham, MA) in HCV patients and healthy controls. For data analysis, standard curve was derived using standard given in the kit and concentration of each cytokine was calculated.

### ELISA

Plasma antibodies including IgG, IgM and IGA were analysed by ELISA (Invitrogen, Waltham, MA), according to the manufacturer’s instructions. In brief, plasma samples were diluted with 1X assay buffer at a ratio of 1: 500,000 for IgG and 1: 100,000 for IgM and IgA detection. Wells were washed twice with 400 µl wash buffer. 100 µl of standards were added, following 100 µl of prediluted samples and 50 µl HRP conjugated antibody. Plate was then sealed and incubated on shaker for at 400 rpm for 1 hr at room temperature. Wells were then washed four times with wash buffer and 100 µl of TMB substrate solution was added and further incubated for 30 min in dark. Reaction was then stopped by adding 100 µl of stop solution. Absorbance reading was taken on a spectrophotometer using 450 nm as primary wavelength and 620 nm as reference wavelength.

### Statistical analysis

Statistical analyses were performed using GraphPad Prism 5.0 software (GraphPad Inc, San Diego, CA, USA). Normal distribution for each parameter was tested with D’Agostino and Pearson omnibus test. To determine the changes in CD4 + CXCR5 + T_FH_, CD4 + CXCR5 + T and B cells in HCV patients pre and post DAA treatment, Paired T test or Wilcoxon matched-pairs signed rank test was used for parametric and nonparametric data, respectively. Comparisons between HC and HCV patients were made using Unpaired T test or Mann–Whitney U test for parametric and nonparametric data, respectively. Correlation significance was analysed with Pearson correlation coefficient. P value < 0.05 was considered for significance.

## Supplementary information

Supplementary file 1

## Data Availability

All the relevant data that supports the findings of this study and information regarding reagents used in this study are available from the corresponding author upon reasonable request.

## References

[1] 1World Health Organization. 2017. Hepatitis C. Available on October 13, 2017. Updated July. Available from: URL: https://www.who.int/mediacentre/factsheets/fs164/en/ [Google Scholar].

[2] Axley P, Ahmed Z, Ravi S, Singal AK (2018). Hepatitis C virus and hepatocellular carcinoma: a narrative review. J. Clin. Transl. Hepatol..

[3] Ashfaq UA, Javed T, Rehman S, Nawaz Z, Riazuddin S (2011). An overview of HCV molecular biology, replication and immune responses. Virol. J..

[4] Yi JS, Cox MA, Zajac AJ (2010). T-cell exhaustion: characteristics, causes and conversion. Immunology.

[5] Kahan SM, Wherry EJ, Zajac AJ (2015). T cell exhaustion during persistent viral infections. Virology.

[6] Wherry EJ, Kurachi M (2015). Molecular and cellular insights into T cell exhaustion. Nat. Rev. Immunol..

[7] Morou A, Palmer BE, Kaufmann DE (2014). Distinctive features of CD4+ T cell dysfunction in chronic viral infections. Curr. Opin. HIV AIDS.

[8] Rodriguez S, Roussel M, Tarte K, Ame-Thomas P (2017). Impact of chronic viral infection on T-cell dependent humoral immune response. Front. Immunol..

[9] Spaan M (2015). CD4+ CXCR5+ T cells in chronic HCV infection produce less IL-21, yet are efficient at supporting B cell responses. J. Hepatol..

[10] MacParland SA (2016). HCV specific IL-21 producing T cells but Not IL-17A producing T cells are associated with HCV viral control in HIV/HCV coinfection. PLoS ONE.

[11] Comarmond C (2017). Direct-acting antiviral therapy restores immune tolerance to patients with hepatitis C virus-induced cryoglobulinemia vasculitis. Gastroenterology.

[12] Oliviero B (2011). Enhanced B-cell differentiation and reduced proliferative capacity in chronic hepatitis C and chronic hepatitis B virus infections. J. Hepatol..

[13] Oliviero B (2015). Skewed B cells in chronic hepatitis C virus infection maintain their ability to respond to virus-induced activation. J. Viral Hepat..

[14] Dai B (2016). Hepatitis C virus upregulates B-cell receptor signaling: a novel mechanism for HCV-associated B-cell lymphoproliferative disorders. Oncogene.

[15] Wang XX (2018). Recovery of natural killer cells is mainly in post-treatment period in chronic hepatitis C patients treated with sofosbuvir plus ledipasvir. World J. Gastroenterol..

[16] Holmes JA (2019). Dynamic changes in innate immune responses during direct-acting antiviral therapy for HCV infection. J Viral Hepat.

[17] Mondelli MU (2015). Direct-acting antivirals cure innate immunity in chronic hepatitis C. Gastroenterology.

[18] Crotty S (2014). T follicular helper cell differentiation, function, and roles in disease. Immunity.

[19] Fukuda T (1997). Disruption of the Bcl6 gene results in an impaired germinal center formation. J. Exp. Med..

[20] Wikenheiser, D. J. & Stumhofer, J. S. ICOS co-stimulation: friend or foe? *Fronti. Immunol.* **7** (2016). 10.3389/fimmu.2016.00304 PMC4979228 27559335

[21] Bossaller L (2006). ICOS deficiency is associated with a severe reduction of CXCR5+CD4 germinal center Th cells. J. Immunol..

[22] Saeidi A (2018). T-cell exhaustion in chronic infections: reversing the state of exhaustion and reinvigorating optimal protective immune responses. Front. Immunol..

[23] Barber DL (2006). Restoring function in exhausted CD8 T cells during chronic viral infection. Nature.

[24] Shi J (2018). PD-1 controls follicular T helper cell positioning and function. Immunity.

[25] Dai YC, Zhong J, Xu JF (2017). Regulatory B cells in infectious disease (review). Mol. Med. Rep..

[26] Yang M, Rui K, Wang S, Lu L (2013). Regulatory B cells in autoimmune diseases. Cell Mol. Immunol..

[27] Sarvaria A, Madrigal JA, Saudemont A (2017). B cell regulation in cancer and anti-tumor immunity. Cell Mol. Immunol..

[28] Wang WW (2015). CD19+CD24hiCD38hiBregs involved in downregulate helper T cells and upregulate regulatory T cells in gastric cancer. Oncotarget.

[29] Wang G (2017). Characteristics of regulatory B cells in patients with chronic hepatitis B virus infection in different immune phases. Discov. Med..

[30] Das A (2012). IL-10-producing regulatory B cells in the pathogenesis of chronic hepatitis B virus infection. J. Immunol..

[31] Nie Y (2004). The role of CXCR4 in maintaining peripheral B cell compartments and humoral immunity. J. Exp. Med..

[32] Raziorrouh B (2016). Virus-specific CD4+ T cells have functional and phenotypic characteristics of follicular T-helper cells in patients with acute and chronic HCV infections. Gastroenterology.

[33] Zhang ZH (2018). Interleukin-7 regulates T follicular helper cell function in patients with chronic hepatitis C. Viral Immunol..

[34] Smits M (2020). Follicular T helper cells shape the HCV-specific CD4+ T cell repertoire after virus elimination. J. Clin. Investig..

[35] Rodrigue-Gervais IG (2010). Dendritic cell inhibition is connected to exhaustion of CD8+ T cell polyfunctionality during chronic hepatitis C virus infection. J. Immunol..

[36] Semmo N, Klenerman P (2007). CD4+ T cell responses in hepatitis C virus infection. World J. Gastroenterol..

[37] Luxenburger H, Neumann-Haefelin C, Thimme R, Boettler T (2018). HCV-specific T Cell responses during and after chronic HCV infection. Viruses.

[38] Radziewicz H (2008). Impaired hepatitis C virus (HCV)-specific effector CD8+ T cells undergo massive apoptosis in the peripheral blood during acute HCV infection and in the liver during the chronic phase of infection. J. Virol..

[39] Terrier B (2012). Interleukin-21 modulates Th1 and Th17 responses in giant cell arteritis. Arthritis Rheum..

[40] Dorner T, Radbruch A (2007). Antibodies and B cell memory in viral immunity. Immunity.

[41] Burton AR (2018). Circulating and intrahepatic antiviral B cells are defective in hepatitis B. J. Clin. Investig..

[42] Baumgarth N (2013). How specific is too specific? B-cell responses to viral infections reveal the importance of breadth over depth. Immunol. Rev..

[43] Chen X, Jensen PE (2008). The role of B lymphocytes as antigen-presenting cells. Arch. Immunol. Ther. Exp. (Warsz).

[44] Tobon GJ, Izquierdo JH, Canas CA (2013). B lymphocytes: development, tolerance, and their role in autoimmunity-focus on systemic lupus erythematosus. Autoimmune Dis..

[45] Moir S, Fauci AS (2013). Insights into B cells and HIV-specific B-cell responses in HIV-infected individuals. Immunol. Rev..

[46] Charles ED, Dustin LB (2009). Hepatitis C virus-induced cryoglobulinemia. Kidney Int..

[47] Dammacco F (2000). The lymphoid system in hepatitis C virus infection: autoimmunity, mixed cryoglobulinemia, and Overt B-cell malignancy. Semin. Liver Dis..

[48] Moir S, Fauci AS (2009). B cells in HIV infection and disease. Nat. Rev. Immunol..

[49] Kohli A (2015). Virological response after 6 week triple-drug regimens for hepatitis C: a proof-of-concept phase 2A cohort study. Lancet.

