# Supplementary material for: Reconstitution of T follicular helper-humoral immune axis with elimination of hepatitis C virus
Source: Sci Rep. 2020 Nov 16;10:19924. doi: 10.1038/s41598-020-77020-2 (PMC7669852; doi:10.1038/s41598-020-77020-2)

**Reconstitution of T follicular helper-humoral immune axis with elimination of hepatitis C virus**

**Authors:** Arshi Khanam^1^, Shyamasundaran Kottilil^1^, Eleanor Wilson^1^

**Affiliation**

1. Division of Clinical Care and Research, Institute of Human Virology, University of Maryland School of Medicine, Baltimore, MD, USA

**Correspondence to:** Eleanor Wilson, M.D., M.H.S.,

Division of Clinical Care and Research,

Institute of Human Virology,

University of Maryland, School of Medicine,

725 West Lombard Street, S218, Baltimore, MD 21201.

E‐mail: [Eleanor.wilson@ihv.umaryland.edu](mailto:Eleanor.wilson@ihv.umaryland.edu)

<Tel:+1-410-706-1710>

**Supplementary Information**

**Supplementary Table: 1 List of antibodies used in this study.**

| Antibody | Fluorochrome | Clone | Catalog no. | Company |
| --- | --- | --- | --- | --- |
| CD3 | AF700 | UCHT1 | 300324 | BioLegend |
| CD4 | PerCP-Cy5.5 | A161A1 | 357414 | BioLegend |
| CXCR5 | BV421 | J252D4 | 356920 | BioLegend |
| CXCR3 | FITC | G025H7 | 353704 | BioLegend |
| CCR6 | APC-Cy7 | [G034E3](https://www.biolegend.com/en-us/search-results?Clone=G034E3) | 353432 | BioLegend |
| CCR7 | BV510 | G043H7 | 353232 | BioLegend |
| PD-1 | PeCy7 | EH12.2H7 | 329918 | BioLegend |
| ICOS | PE | ISA3 | 12-9948-42 | e Bioscience |
| CD45RA | BV605 | HI100 | 304134 | BioLegend |
| BCL6 | APC | 7D1 | 358506 | BioLegend |
| CD19 | FITC | 2185634 | 555412 | BD Pharmingen |
| CD24 | BV605 | ML5 | 311124 | BioLegend |
| CD27 | BV510 | L218 | 563090 | BD Biosciences |
| CD38 | PerCP-Cy5.5 | HB7 | 356613 | BioLegend |
| IgD | AF700 | IA62 | 348229 | BioLegend |
| CXCR4 | APC-Cy7 | 12G5 | 306528 | BioLegend |
| BLIMP-1 | APC | ACTU0116021 | IC36081a | R&D systems |
| IL-17A | PE | BL168 | 512306 | BioLegend |
| IL-21 | AF647 | 3A3-N2 | 513005 | BioLegend |
| IL-22 | PeCy7 | 2G12A41 | 366707 | BioLegend |
| IFN-γ | BV605 | 4S.B3 | 502536 | BioLegend |
| TFN-α | BV510 | Mab11 | 502950 | BioLegend |

**Supplementary Figure**

**A Figure 1**


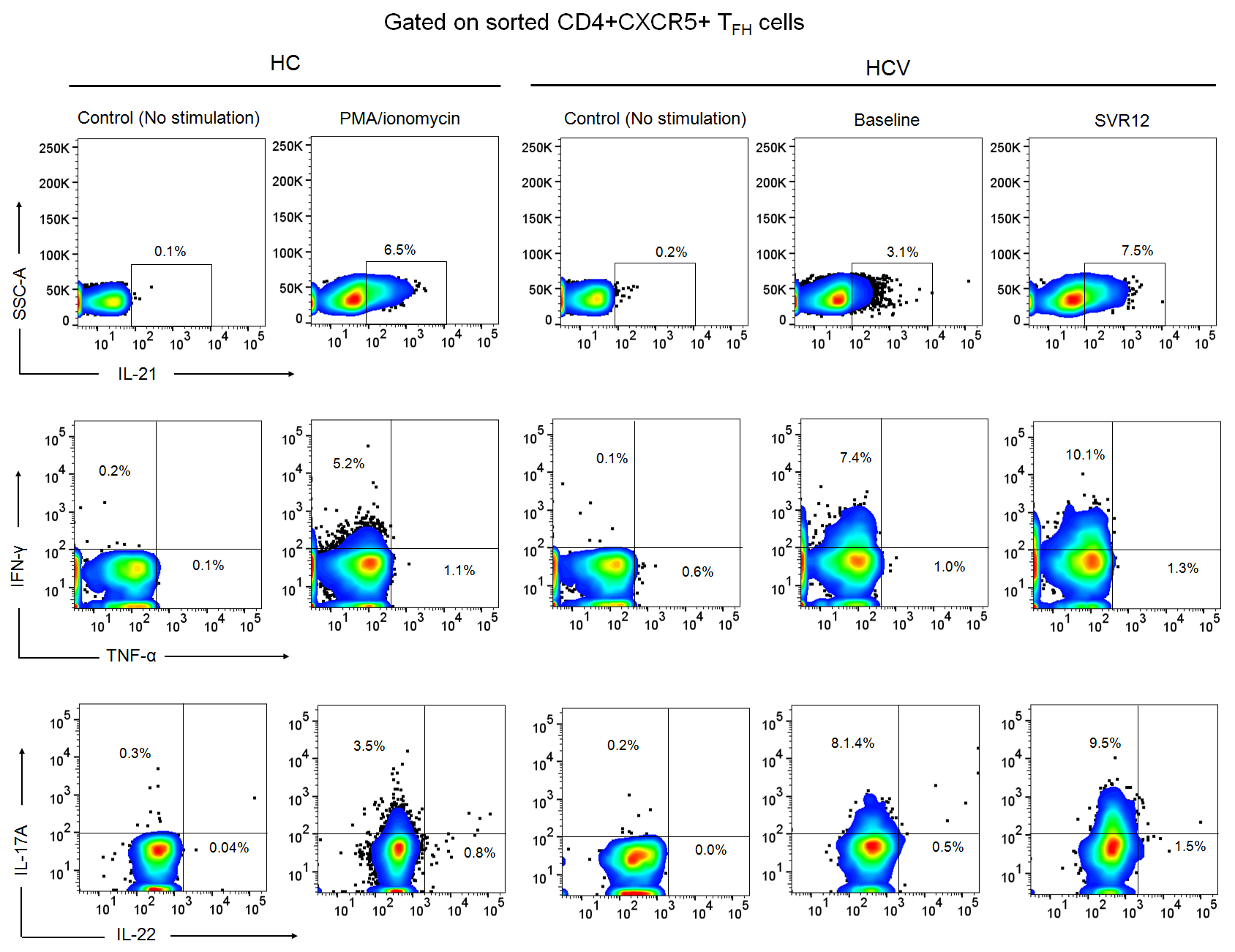


Supplementary figure 1: Global IL-21 secretion enhanced post DAA therapy. To measure global cytokine production, CD3+CD4+CXCR5+ T_FH_ cells were sorted from HC (n=5) and HCV patients (n=5), stimulated with PMA/ionomycin for 18 hrs and cytokine production was analysed by flow cytometry. (A) Representative flow cytometry images represents IL-21, IFN-γ, TNF-α, IL-17A and IL-22 production by sorted T_FH_ cells. (B) Cumulative data has been presented in line graphs for HCV patients and scatter dot plot for HC. Comparisons between HC and HCV patients were evaluated by unpaired T test or Mann Whitney test. For analysing data in HCV patients pre and post DAA therapy, Paired T test or Wilcoxon matched-pairs signed rank test was used.

**B**


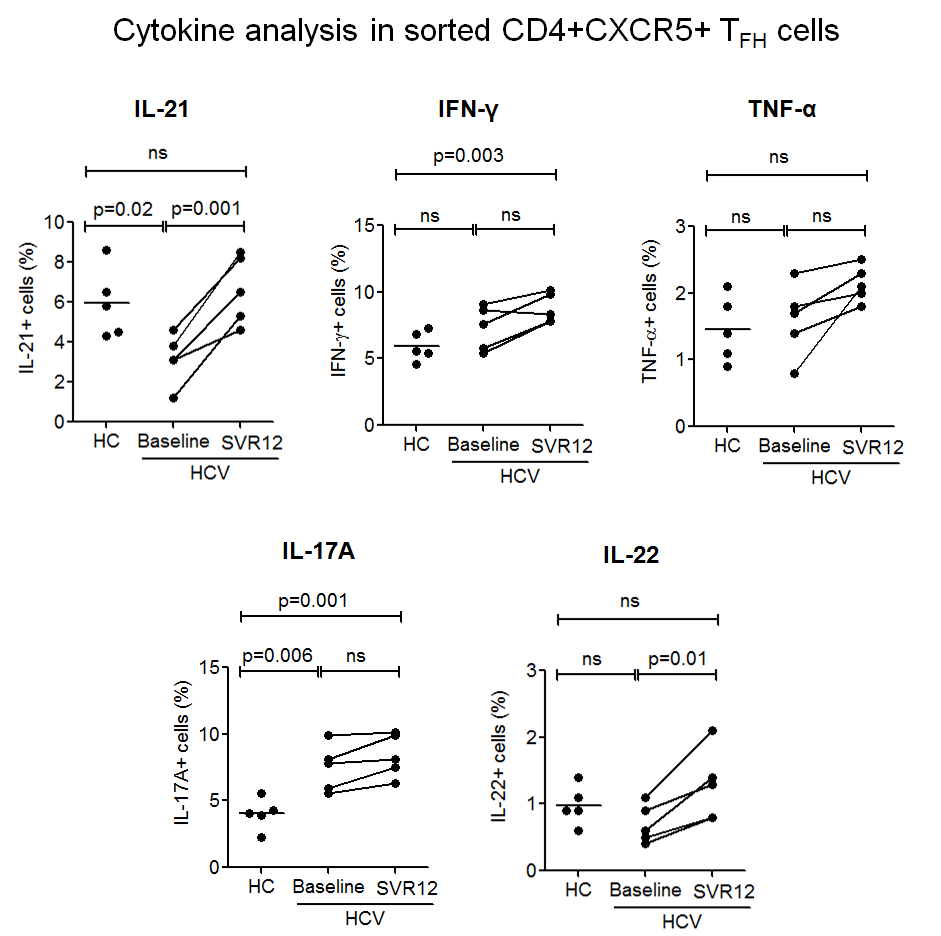

Supplement: Supplementary file 1 [file 41598_2020_77020_MOESM1_ESM.docx]
